# Short-term outcome in robotic vs laparoscopic and open rectal tumor surgery within an ERAS protocol: a retrospective cohort study from the Swedish ERAS database

**DOI:** 10.1007/s00464-021-08486-y

**Published:** 2021-04-15

**Authors:** Daniel Asklid, Olle Ljungqvist, Yin Xu, Ulf O. Gustafsson

**Affiliations:** 1 Department of Clinical Sciences, Division of Surgery, Danderyd Hospital, Karolinska Institutet, 18288 Stockholm, Danderyd Sweden; 2 Department of Surgery, Örebro & Institute of Molecular Medicine and Surgery, Örebro University and University Hospital, Karolinska Institutet, Stockholm, Sweden; 3 Clinical Epidemiology and Biostatistics, School of Medical Sciences, Örebro University, Örebro, Sweden

**Keywords:** Robotic surgery, Rectal tumor, ERAS

## Abstract

**Background:**

Advantages of robotic technique over laparoscopic technique in rectal tumor surgery have yet to be proven. Large multicenter, register-based cohort studies within an optimized perioperative care protocol are lacking. The aim of this retrospective cohort study was to compare short-term outcomes in robotic, laparoscopic and open rectal tumor resections, while also determining compliance to the enhanced recovery after surgery (ERAS)®Society Guidelines.

**Methods:**

All patients scheduled for rectal tumor resection and consecutively recorded in the Swedish part of the international ERAS® Interactive Audit System between January 1, 2010 to February 27, 2020, were included (*N* = 3125). Primary outcomes were postoperative complications and length of stay (LOS) and secondary outcomes compliance to the ERAS protocol, conversion to open surgery, symptoms delaying discharge and reoperations. Uni- and multivariate comparisons were used.

**Results:**

Robotic surgery (*N* = 827) had a similar rate of postoperative complications (Clavien–Dindo grades 1–5), 35.9% compared to open surgery (*N* = 1429) 40.9% (OR 1.15, 95% CI (0.93, 1.41)) and laparoscopic surgery (*N* = 869) 31.2% (OR 0.88, 95% CI (0.71, 1.08)). LOS was longer in the open group, median 9 days (IRR 1.35, 95% CI (1.27, 1.44)) and laparoscopic group, 7 days (IRR 1.14, 95% CI (1.07, 1.21)) compared to the robotic group, 6 days. Pre- and intraoperative compliance to the ERAS protocol were similar between groups.

**Conclusions:**

In this multicenter cohort study, robotic surgery was associated with shorter LOS compared to both laparoscopic and open surgery and had lower conversion rates vs laparoscopic surgery. The rate of complications was similar between groups.

During the last decades, immense technical developments have been made in colorectal surgery. Previous studies show that the laparoscopic technique improve short-term outcomes in colorectal surgery compared to open technique with comparable oncological outcome [1–4]. In rectal procedures, however, the narrow pelvis makes surgery difficult and advantages in favor of laparoscopy are less evident [5].

The robotic platform may have advantages over laparoscopic techniques in rectal surgery because of superior three-dimensional view, more correct ergonomic position for the surgeon and most likely a shorter learning curve compared to the laparoscopic approach [6–9].

Although a few retrospective cohort studies indicate improved short-term outcomes in favor of robotic rectal surgery [10, 11], the majority of randomized controlled trials (RCTs) have been underpowered and meta-analyses have not been able to confirm these results [6, 8, 12, 13]. Since higher costs have been reported for robotic surgery compared with laparoscopic and open surgery [13, 14] comparative studies with larger sample sizes that also reflect clinical reality are necessary, in order to better justify its use.

The international ERAS®Society Interactive Audit System (EIAS) is a database [15] containing more than 80,000 consecutively recorded patients, each with more than 300 recorded variables, including compliance measures to all perioperative interventions recommended by the ERAS®Society Guidelines. EIAS is used for implementation of the ERAS®Society Guidelines and then used to sustain these principles of care and represents a source for comparing surgical techniques while also controlling for other key care items that may impact clinical outcomes. All patients recorded in the database are treated with aim of using the same evidence-based protocol including 24 interventions, resulting in improved recovery, reduced morbidity rates and shortened LOS after colorectal surgery [16–18]. The perioperative period and outcome from surgery can be analyzed and compared in detail and be related to other variables that may impact the main clinical outcomes.

The current study aims to compare short-term outcome in patients operated on with robotic, laparoscopic and open rectal surgery, while controlling for compliance to the ERAS protocol using data from the Swedish part of the international EIAS database.

## Materials and methods

### Study design and setting

Out of 36 hospitals performing colon or rectal tumor surgery in Sweden, 18 hospitals including five of the seven university hospitals, are currently recording clinical data in the Web-based International ERAS® Interactive Audit System. All units aim to treat their patients according the same perioperative ERAS protocol with 24 evidence-based perioperative pre-, intra and postoperative interventions [17]. Clinical data, including more than 300 variables on protocol adherence and clinical outcomes are prospectively and consecutively recorded in the system.

The Swedish part of the international database containing more than 13,000 patients was validated in the end of 2019 according to coverage of patients, accuracy of data, and rate of missing values (data not yet published). The first hospitals have been recording patients in the database since 2010, with increasingly more centers joining over the years. The most recent joined in April 2017.

In the present retrospective cohort study, we aimed to investigate potential differences in short-term outcome and compliance to the ERAS protocol in patients with rectal tumor (benign or malignant) operated with robotic, laparoscopic or open approach. All data from patients operated in Sweden with either anterior resection (AR) or abdominoperineal resection (APR) recorded between January 1, 2010 to February 27, 2020 were collected from EIAS and analyzed.

This study was approved by the Regional Ethical Review Board in Stockholm (2020–00,435) and performed in accordance with the Declaration of Helsinki of the World Medical Association (1989) and is reported according to the criteria set out in the Strengthening the Reporting of Observational studies in Epidemiology (STROBE) checklist [19].

### Participants

A total of 3125 patients, representing all recorded elective patients in the Swedish part of the EIAS with benign or malignant rectal tumor operated with robotic, laparoscopic or open rectal AR or APR surgery, were included in the study. A rectal tumor was defined as a lesion situated within 15 cm from anal verge, estimated by rigid rectoscopy. The study cohort consists of all stages of rectal cancer. Patients operated on with emergency surgery were not recorded in the database, and hence not included in the study.

Data on basic characteristics, pre-, intra- and postoperative compliance to ERAS interventions and outcome from surgery were retrieved from the EIAS for all patients.

### Outcome variables

Primary outcomes were 30-day complications, both surgical and non-surgical (Clavien I–II, > III) [20] and LOS (postoperative nights). Secondary outcomes: compliance to the ERAS protocol for pre- and intraoperative elements [21], conversions to open surgery, symptoms delaying discharge (urinary retention, nausea or vomiting, obstipation or diarrhea, paralytic ileus and pain), reoperations (30-day) and duration of surgery (hours).

Criteria for discharge was defined as no complications requiring further hospitalization, return of bowel function defined as passage of flatus or stool, > 6 h mobilization out of bed and pain controlled with oral analgesics. Operating time included all preparations for the minimal invasive approaches as well as docking the robot. Conversion to open surgery means that the operation is changed from using minimally invasive techniques and instrumentation to a classic open operation. In addition, there were no laparoscopically assisted operations in this study, only conversions to open surgery.

### Exposure variables

Exposure variable was surgical approach. The robotic group (reference group) was compared to the laparoscopic and open group regarding basic characteristics, intraoperative variables, compliance to the ERAS protocol and outcome from surgery. All patients were analyzed according to intention to treat.

### Potential confounders

Adjustment variables were gender (male or female), age (26–50 years, 51–75 years and 76–100 years), body mass index (BMI, underweight 15 to < 18.5, normal weight 18.5 to < 25, overweight 25 to < 30, obese > 30), American Society of Anesthesiologists (ASA, class 1, 2 and > 3) physical status classification, surgical procedure (AR or APR), year of surgery (2010–2015 and 2016–2020), alcohol abuse (yes, no or stopped due to surgery), pre- and intraoperative compliance rate, and other six binary variables (yes or no), including additional surgical procedure, previous surgery to the abdomen, preoperative chemotherapy, preoperative radiotherapy, severe pulmonary disease and cancer.

### Data analysis

A power analysis was made on primary outcome (LOS) on an estimated detectable difference of mean two days in favor of robotic compared to laparoscopic rectal surgery [11]. With 80% power at a two-sided alpha of 0.05, the number of patients was estimated to 38 in each group (calculated on a two-group comparison).

To test if the three surgical approaches (robotic surgery was the reference group) differed in basic characteristics (Table 1), pre- and intraoperative compliance (Table 2), postoperative compliance (Table 3), and symptoms delaying discharge, univariate regressions were performed (logistic regression, ordinal logistic regression, linear regression, and non-parametric Kruskal–Wallis test for binary variables, ordinal variables, continuous variables with the normal distribution, and continuous variables without the normal distribution, respectively). Normal distribution was tested using Shapiro–Francia test. No evidence of violation of the proportional odds assumption was found using Brant test. Compliance data were calculated as the numbers of achieved interventions divided with the total number of pre- and postoperative interventions excluding any non-applicable interventions. The postoperative ERAS items of the ERAS protocol were not included in the analysis of adherence since they also could be regarded as outcome variables.

**Table 1:** Basic characteristics stratified by surgical approach

	Surgical approach			Group comparison^a^	
	Open (N = 1429)	Laparoscopic (N = 869)	Robotic (N = 827)	Open vs. robotic *p* value	Laparoscopic vs. robotic *p* value
Sex				0.242	0.722
Male	867 (60.7)	498 (57.3)	481 (58.2)		
Female	562 (39.3)	371 (42.7	346 (41.8)		
Missing	0 (0)	0 (0)	0 (0)		
Age group				0.329	0.132
20–50	92 (6.4)	57 (6.6)	61 (7.4)		
51–75	942 (65.9)	557 (64.1)	550 (66.5)		
76–100	394 (27.6)	254 (29.2)	216 (26.1)		
Missing	1 (0.1)	1 (0.1)	0 (0)		
Cancer				0.012	0.384
No	37 (2.6)	48 (5.5)	38 (4.6)		
Yes	1392 (97.4)	821 (94.5)	789 (95.4)		
Missing	0 (0)	0 (0)	0 (0)		
Procedure type				0.043	0.648
Anterior resection	849 (59.4)	563 (64.8)	527 (63.7)		
Rectum amputation	580 (40.6)	306 (35.2)	300 (36.3)		
Missing	0 (0)	0 (0)	0 (0)		
Additional procedures				< 0.001	0.255
No	625 (43.7)	671 (77.2)	769 (93.0)		
Yes	158 (11.1)	50 (5.8)	45 (5.4)		
Missing	646 (45.2)	148 (17.0)	13 (1.6)		
Smoking				0.080	0.682
No	1233 (86.3)	761 (87.6)	714 (86.3)		
Stopped due to surgery	41 (2.9)	34 (3.9)	21 (2.5)		
Yes	100 (7.0)	37 (4.3)	41 (5.0)		
Missing	55 (3.8)	37 (4.2)	51 (6.2)		
Alcohol				0.007	0.003
No	684 (47.9)	551 (63.4)	563 (68.1)		
Stopped due to surgery	10 (0.7)	81 (9.3)	21 (2.5)		
Yes	40 (2.8)	30 (3.5)	49 (5.9)		
Missing	695 (48.6)	207 (23.8)	194 (23.5)		
Previous surgery to the abdominal region				0.020	0.370
No	1029 (72.0)	644 (74.1)	636 (76.9)		
Yes	386 (27.0)	211 (24.3)	188 (22.7)		
Missing	14 (1.0)	14 (1.6)	3 (0.4)		
Diabetes				0.569	0.704
No	1222 (85.5)	754 (86.8)	714 (86.3)		
Yes	206 (14.4)	112 (12.9)	112 (13.5)		
Missing	1 (0.1)	3 (0.3)	1 (0.2)		
BMI				0.408	0.573
Under weight	28 (2.0)	15 (1.7)	18 (2.2)		
Normal weight	605 (42.3)	360 (41.5)	320 (38.7)		
Obese	549 (38.4)	346 (39.8)	340 (41.1)		
Over weight	233 (16.3)	135 (15.5)	129 (15.6)		
Missing	14 (1.0)	13 (1.5)	20 (2.4)		
ASA physical status^b^				0.584	< 0.001
1	220 (15.4)	191 (22.0)	127 (15.4)		
2	787 (55.1)	509 (58.6)	498 (60.2)		
3	392 (27.4)	155 (17.8)	186 (22.5)		
4	19 (1.3)	3 (0.4)	0 (0.0)		
5	1 (0.1)	0 (0.0)	0 (0.0)		
Missing	10 (0.7)	11 (1.2)	16 (1.9)		
Severe pulmonary disease				0.204	0.065
No	676 (47.3)	645 (74.2)	795 (96.1)		
Yes	24 (1.7)	27 (3.1)	19 (2.3)		
Missing	729 (51.0)	197 (22.7)	13 (1.6)		
Preop chemotherapy				0.089	< 0.001
No	1170 (81.9)	828 (95.3)	700 (84.6)		
Yes	254 (17.8)	39 (4.5)	124 (15.0)		
Missing	5 (0.3)	2 (0.2)	3 (0.4)		
Preop radiotherapy				0.036	0.001
No	591 (41.4)	470 (54.1)	381 (46.1)		
Yes	832 (58.2)	396 (45.6)	446 (53.9)		
Missing	6 (0.4)	3 (0.3)	0 (0.0)		
Diverting ileostomy				0.350	0.539
No	890 (62.3)	537 (61.8)	499 (60.3)		
Yes	538 (37.6)	332 (38.2)	328 (39.7)		
Missing	1 (0.1)	0 (0.0)	0 (0.0)		

Values in parenthesis are percentages if not stated otherwise

*ASA* American Society of Anesthesiologists physical status, *BMI* Body Mass Index

^a^Univariate regression, each variable listed in the table regressed on surgical approach. Robotic surgery was the reference group

For binary variables univariate logistic regression was performed. For ordinal variables univariate ordinal logistic regression was performed

^b^For ASA physical status, since there are cell sizes equal to zero, ASA class 3–5 were combined into one group

**Table 2:** Pre- and intraoperative compliance stratified by surgical approach

	Surgical approach			Group Comparison^a^	
	Open (N = 1429)	Laparoscopic (N = 869)	Robotic (N = 827)	Open vs. Robotic *p* value	Laparoscopic vs. Robotic *p* value
*Preoperative compliance*					
Preadmission education given				0.016	0.438
Non-compliant	77 (5.4)	22 (2.5)	26 (3.2)		
Compliant	1348 (94.3)	845 (97.2)	795 (96.1)		
Missing	4 (0.3)	2 (0.3)	6 (0.7)		
Preop oral carbohydrate treatment				0.014	0.365
Non-compliant	85 (6.0)	39 (4.5)	30 (3.6)		
Compliant	1288 (90.1)	807 (92.9)	777 (94.0)		
Missing	56 (3.9)	23 (2.6)	20 (2.4)		
Oral bowel preparation				0.147	0.002
Non-compliant	126 (8.8)	29 (3.2)	50 (6.1)		
Compliant	646 (45.2)	415 (47.8)	333 (40.3)		
Missing	8 (0.6)	3 (0.4)	10 (1.2)		
Preop long-acting sedative medication				< 0.001	< 0.001
Non-compliant	152 (10.6)	96 (11.0)	179 (21.6)		
Compliant	1230 (86.1)	746 (85.9)	620 (75.0)		
Missing	47 (3.3)	27 (3.1)	28 (3.4)		
Antibiotic prophylaxis before incision				0.132	0.411
Non-compliant	16 (1.1)	7 (0.8)	4 (0.5)		
Compliant	1411 (98.8)	857 (98.6)	821 (99.3)		
Missing	2 (0.1)	5 (0.6)	2 (0.2)		
Thrombosis prophylaxis				0.469	0.036
Non-compliant	59 (4.1)	16 (1.8)	29 (3.5)		
Compliant	1368 (95.7)	849 (97.7)	795 (96.1)		
Missing	2 (0.2)	4 (0.5)	3 (0.4)		
PONV prophylaxis administered				< 0.001	0.268
Non-compliant	54 (3.8)	16 (1.8)	11 (1.3)		
Compliant	530 (37.1)	345 (39.7)	369 (44.6)		
Missing	7 (0.5)	3 (0.4)	4 (0.5)		
*Intraoperative compliance*					
Infusion of vasoactive drugs				0.141	0.215
Non-compliant	327 (22.9)	198 (22.8)	174 (21.0)		
Compliant	1033 (72.3)	632 (72.7)	643 (77.8)		
Missing	69 (4.8)	39 (4.5)	10 (1.2)		
Upper-body forced-air heating cover used				0.068	< 0.001
Non-compliant	18 (1.3)	50 (5.8)	19 (2.3)		
Compliant	1378 (96.4)	800 (92.0)	793 (95.9)		
Missing	33 (2.3)	19 (2.2)	15 (1.8)		
Total IV volume of fluids intraoperatively				< 0.001	0.890
Non-compliant	139 (9.7)	22 (2.5)	29 (3.5)		
Compliant	1290 (90.3)	847 (97.5)	798 (96.5)		
Missing	0 (0)	0 (0)	0 (0)		
Preoperative compliance rate (%)				0.816	< 0.001
N	1312	809	762		
Mean (SD)	93.45 (10.00)	95.85 (8.59)	93.47 (9.44)		
Missing	117 (8.2)	60 (6.9)	65 (7.9)		
Intraoperative compliance rate (%)				< 0.001	0.039
N	1335	816	806		
Mean (SD)	88.11 (16.98)	89.09 (18.52)	90.86 (16.30)		
Missing	94 (6.6)	53 (6.1)	21 (2.5)		
Pre- and intraoperative compliance rate combined (%)				0.162	0.001
N	1242	768	747		
Mean(SD)	91.69 (8.62)	93.83 (8.27)	92.56 (7.64)		
Missing	187 (13.1)	101 (11.6)	80 (9.7)		

Values in parenthesis are percentages if not stated otherwise

^a^Univariate regression, each variable listed in the table regressed on surgical approach. Robotic surgery was the reference group

For all variables listed in the table except compliance rates, univariate logistic regression was performed. For intraoperative compliance rate, linear regression was performed since intraoperative compliance is normally distributed (*p* = 0.095 for the Shapiro–Francia test). For preoperative compliance rate, and pre- and intraoperative compliance rates combined, non-parametric Kruskal–Wallis test was performed since they are not normally distributed (all *p* < 0.001)

**Table 3:** Postoperative compliance stratified by surgical approach

	Surgical approach			Group comparison^a^	
	Open (*N* = 1429)	Laparoscopic (*N* = 869)	Robotic (*N* = 827)	Open vs. Robotic *p* value	Laparoscopic vs. robotic *p* value
Total IV volume of fluids day 0 (mL)				< 0.001	0.087
N	1429	869	827		
Mean (SD)	3859.07 (2047.22)	2548.26 (1494.55)	2606.11 (1385.25)		
Missing	0 (0)	0 (0)	0 (0)		
Time to passage of flatus (days)				< 0.001	0.002
N	1209	723	705		
Mean (SD)	2.04 (2.48)	1.44 (1.79)	1.60 (1.67)		
Missing	220 (15.4)	146 (16.8)	122 (14.8)		
First passage of stool (days)				< 0.001	0.003
N	1357	824	779		
Mean (SD)	3.13 (3.65)	2.33 (3.61)	2.50 (2.84)		
Missing	72 (5.0)	45 (5.2)	48 (5.8)		
Time to tolerating solid food (days)				< 0.001	< 0.001
N	1273	728	753		
Mean (SD)	4.09 (6.13)	2.81 (4.88)	2.88 (3.45)		
Missing	156 (10.9)	141 (16.2)	74 (9.0)		
Termination of urinary drainage (days)				< 0.001	< 0.001
N	1151	703	713		
Mean (SD)	6.38 (5.54)	4.63 (6.07)	4.55 (10.63)		
Missing	278 (19.5)	166 (19.1)	114 (13.8)		
Time to pain control with oral analgesics (days)				< 0.001	< 0.001
N	1356	775	799		
Mean (SD)	5.23 (4.07)	3.58 (4.56)	2.85 (4.04)		
Missing	73 (5.1)	94 (10.8)	28 (3.4)		

Values in parenthesis are percentages if not stated otherwise

^a^For each variable listed in the table a non-parametric Kruskal–Wallis test was performed to test the surgical approach differences since they are not normally distributed based on Shapiro–Francia test (all *p* < 0.001). Robotic surgery was the reference group

Multivariate analyses were further performed to test the association between short-term outcomes and surgical approach with adjustment for confounders (zero-truncated negative binomial regression for length of stay, logistic regression for complication, symptoms delaying discharge, reoperation and conversion to open surgery, while linear regression for duration of operation). Conversions from laparoscopic/robotic surgery to open surgery were analyzed on intention-to-treat basis. Box-cox transformation toward a normal distribution was performed for duration of surgery (power = 0.5) before regression. The variables included in the multivariable analyses had 0.1% – 35.1% missing information. This was handled via multiple imputation using iterative chained equations. Thirty-six imputations were created to match the percentage of missing data [22].

Continuous variables were presented as mean with standard deviation (SD) or median (interquartile range) when appropriate. Categorical variables were presented as frequencies and percentage.

A *p* value < 0.05 or 95% confidence interval (CI) not including 1 was considered statistically significant. Stata version 16.0 (StataCorp, College Station, Texas, United States of America) was used for statistical analysis.

## Results

### Surgical approach

Out of altogether 3125 patients included in the study, 827 (26.5%) had robotic rectal surgery, 869 (27.8%) were treated laparoscopically and 1429 (45.7%) had open surgery, Table 1.

### Time and distribution of procedures

**Fig. 1:**
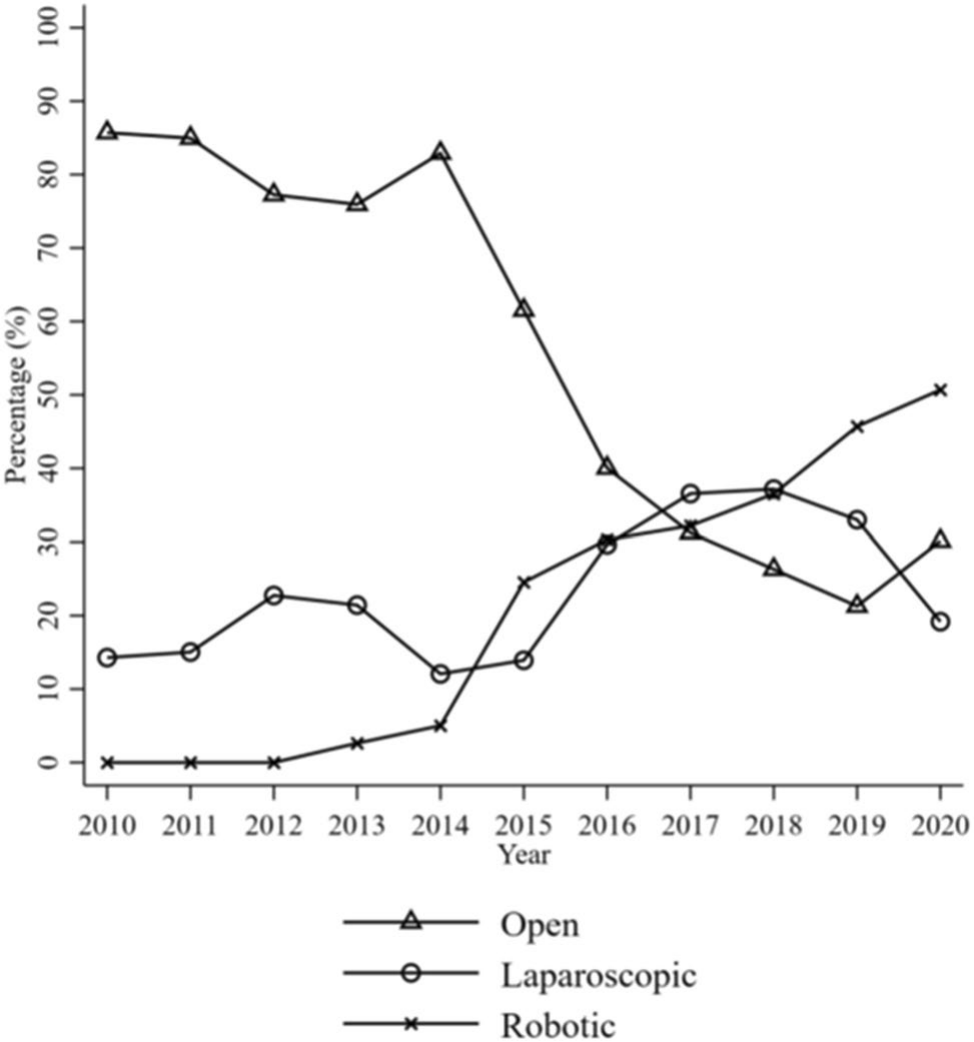
Surgical approach stratified by time. From the year 2015, the rate of open procedures decreased steadily. No robotic procedures were performed before 2013 and the proportion was increasing with time, c2(14) = 753.54, *p* < 0.001

In Fig. 1, the proportion of surgical approaches are shown in relation to time. During the early years in the study period, no robotic, but a high rate of open surgery procedures were performed. Toward the end of the time period, this proportion had been reversed. The proportion of different approaches stratified by hospitals was unevenly distributed, partly due to the fact that some hospitals lack the robotic platform (data not shown).

### Basic characteristics

Basic characteristics stratified by surgical approach are demonstrated in Table 1. The open surgery group had a small, but significantly higher proportion of cancer patients compared to the robotic group (97.4% vs 95.4%, *p* = 0.012). Fewer APRs were conducted in the robotic group vs open surgery group (36.3% vs 40.6%, *p* = 0.043). Additional procedures were more often performed in the open group (11.1% vs 5.4%, *p* < 0.001) compared to robotic group. The proportion of patients who had previous surgery to the abdomen was larger in the open group compared to robotic group (27.0% vs 22.7%, *p* = 0.020). Both preoperative chemotherapy and radiotherapy were more common in robotic surgery compared to laparoscopic surgery (15.0% vs 4.5%, *p* < 0.001 and 53.9% vs 45.6%, *p* < 0.001). Preoperative radiotherapy was more common in open surgery compared to the robotic group (58.2% vs 53.9%, *p* = 0.036).

Placement of a diverting ileostomy did not differ between groups, Table 1.

### Compliance to the ERAS protocol

Pre- and intraoperative compliance are shown in Table 2. Overall there were very small, but significant, differences in pre- and intraoperative compliance when comparing robotic to laparoscopic surgery (92.6% vs 93.8%, Cohen’s d = 0.16, *p* = 0.001) respectively. Regarding postoperative compliance, minimal invasive surgery showed better results than open surgery in every aspect, and in time to pain control with oral analgesics, robotic surgery was significantly better than both laparoscopic and open surgery, Table 3.

### Short-term outcome—duration of surgery, conversion to open surgery, reoperations, complications, symptoms delaying discharge and LOS

The duration of surgery was shorter among patients operated with open surgery and slightly shorter in laparoscopic surgery compared to robotic surgery, Table 4.

**Table 4:** Short-term outcomes stratified by surgical approach and regression analysis for short-term outcomes

	Surgical approach			Univariate analysis	Multivariate analysis
	Open (*N* = 1429)	Laparoscopic (*N* = 869)	Robotic (*N* = 827)	Open vs Robot / Lap vs Robot	Open vs Robot / Lap vs Robot
Complications at all N (%)				1.24 (1.04, 1.48) / 0.82 (0.67, 1.00)	1.15 (0.93, 1.41) / 0.88 (0.71, 1.08)
No	829 (58.0)	588 (67.7)	523 (63.2)		
Yes	585 (40.9)	271 (31.2)	297 (35.9)		
Missing	15 (1.1)	10 (1.1)	7 (0.9)		
Complications at all N (%)					
No	829 (58.0)	588 (67.7)	523 (63.2)		
Minor complications	181 (12.7)	111 (12.8)	163 (19.7)		
Major complications	117 (8.2)	99 (11.4)	116 (14.0)		
Missing	302 (21.1)	71 (8.1)	25 (3.1)		
Conversion to open surgery N (%)				− / 2.40 (1.78, 3.25)	− / **2.58 (1.85, 3.60)**
No	1429 (100.0)	713 (82.1)	758 (91.7)		
Yes	0 (0)	156 (17.9)	69 (8.3)		
Missing	0 (0)	0 (0)	0 (0)		
Reoperation N (%)				1.07 (0.82, 1.39) / 0.95 (0.71, 1.28)	1.01 (0.75, 1.37) / 0.99 (0.73, 1.35)
No	1201 (84.1)	761 (87.6)	718 (86.8)		
Yes	172 (12.0)	99 (11.4)	97 (11.7)		
Missing	56 (3.9)	9 (1.0)	12 (1.5)		
Symptoms delaying discharge N (%)				1.39 (1.13, 1.69) / 0.91 (0.72, 1.15)	**1.62 (1.29, 2.04)** / 0.97 (0.76, 1.23)
No	1021 (71.5)	689 (79.3)	642 (77.6)		
Yes	405 (28.3)	480 (20.7)	183 (22.2)		
Missing	3 (0.2)	0 (0)	2 (0.2)		
Total length of stay (days)				1.41 (1.32, 1.52) / 1.12 (1.03, 1.21)	**1.35 (1.27, 1.44)** / **1.14 (1.07, 1.21)**
N	1407	855	823		
Mean (SD)	10.93 (7.07)	8.60 (6.72)	7.82 (6.58)		
Median (interquartile range)	9 (6)	7 (5)	6 (5)		
Missing N (%)	22 (1.5)	14 (1.6)	4 (0.5)		
Duration of surgery (hours)				− 0.21 (-0.24, -0.17) / -0.06 (-0.10, -0.03)	− **0.21 (-0.24, −0.17)** / **−0.05 (−0.08, −0.01)**
N	1413	859	817		
Mean (SD)	4.84 (1.79)	5.49 (1.83)	5.77 (1.91)		
Missing N (%)	16 (1.1)	10 (1.2)	10 (1.2)		

Minor complications = Clavien–Dindo grade I–II. Major complications = Clavien–Dindo grade III–V. Odds ratio and 95% confidence interval (CI) were reported for complications, conversion to open surgery, reoperation and symptoms delaying discharge. Incidence risk ratio and 95% CI were reported for length of stay. Linear coefficient and 95% CI were reported for duration of surgery. Robotic surgery = reference category

Conversion to open surgery was more common in the laparoscopic group (18.0%) compared to the robotic group (8.3%), OR 2.58, 95% CI (1.85, 3.60). No significant differences in the rate of reoperations between groups were found, Table 4.

**Fig. 2:**
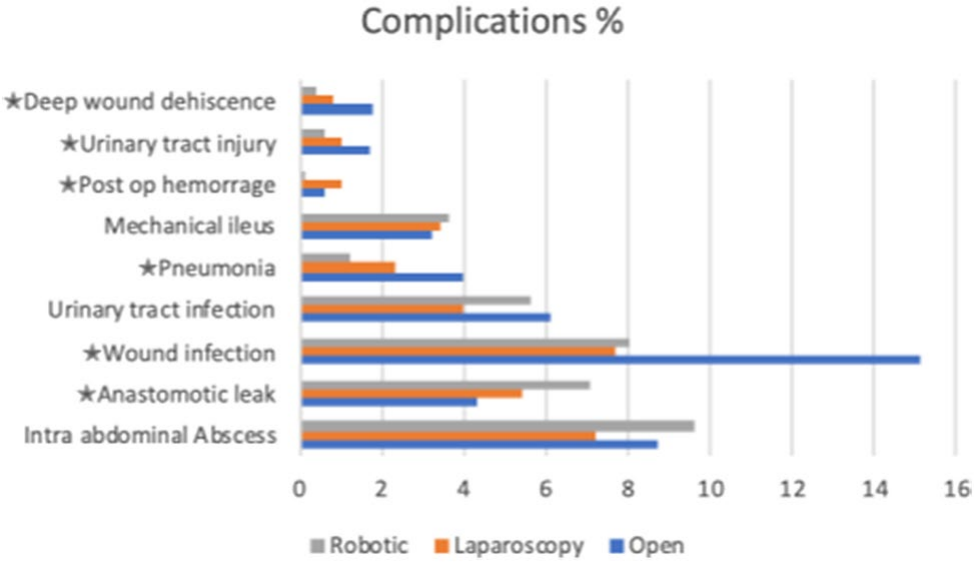
Selected complications (%) stratified by surgical approach. *P value less than 0.05 was considered being significant. In univariate analysis, the rate of deep wound dehiscence (1.8% vs 0.4%), urinary tract injury (1.7% vs 0.6%), pneumonia (4.0% vs 1.2%) and wound infection (15.1% vs 8.0%) was significantly higher in open surgery compared to robotic surgery. Anastomotic leaks however, had a significantly lower rate (4.3% vs 7.1%). Except from a significantly higher rate of postoperative bleeding in the laparoscopic group (1% vs 0.1%) compared to the robotic group no other differences were found. Further 14 complications were compared (not shown) without significant difference between groups with the exception of a significantly higher rate of cardiac arrythmia in the open compared with the robotic group

In univariate analysis, the proportion of patients with any complication indicated a slight difference to the disadvantage for open surgery compared to laparoscopic and robotic surgery (40.9% vs 31.2% and 35.9% respectively), but after adjustment for confounding variables, no difference was found between groups, Table 4. Detailed information on complications are shown in Fig. 2. The proportion of patients having any symptom delaying discharge was 22.1%, 20.7% and 28.3% in the robotic, laparoscopic and open group respectively. After regression analysis the adjusted risk of having symptom delaying discharge was higher in the open group OR 1.62 (1.29, 2.04) but not significantly lower OR 0.97 (0.76, 1.23) in the laparoscopic compared to the robotic group. LOS was median 9 days, 7 days and 6 days in the open, laparoscopic and robotic respectively, this significant difference remained after adjustment for confounding, Table 4. Sensitivity analysis excluding patients with benign disease did not alter the results.

## Discussion

In this study, the largest published cohort comparing short-term outcome in patients undergoing robotic, laparoscopic or open rectal surgery due to rectal tumor, robotic surgery showed better results compared to laparoscopic surgery in terms of rate of conversion to open surgery and LOS. No significant differences were seen in overall complication rates, reoperations or symptoms delaying discharge in multivariate analysis. Although pre- and intraoperative compliance to the ERAS protocol was high and similar between groups, almost all postoperative enhanced recovery measures were worse in open surgery compared to minimal invasive surgery.

Ever since the introduction of total mesorectal excision (TME) in open rectal cancer surgery, further improvements of outcome have been attempted with enhanced recovery protocols and minimal invasive surgical techniques. Laparoscopic colorectal surgery was introduced in the 1990s [23], but it took a long time before short-term outcomes [1, 3] were proven better than open surgery. Although similar oncological outcomes have been reported [1, 3, 24] in most studies, the debate is still ongoing since two recent reports indicate worse results in laparoscopy vs open rectal cancer surgery using a pathologic composite score [25, 26].

In 2006, the first publication on robotic total mesorectal excision (TME) for rectal cancer was published [27]. This caught large interest, since some of the potential disadvantages reported using traditional laparoscopy in pelvic surgery include poor vision in the narrow pelvic cavity, limited dexterity, unstable instruments, poor ergonomic set up and a proposed longer learning curve [5, 27]. Robotic rectal surgery has since been widely introduced, to a great extent because of its potential to overcome many of the shortcomings of traditional laparoscopy in the pelvis using 3-dimensional vision, stable camera, endowristed instruments and eliminated tremor [7, 8]. The rapid implementation has though not been matched by convincing results in favor of robotic surgery. Although most studies so far investigating and comparing outcomes from laparoscopic and robotic rectal surgery are hampered by small sample sizes, a recent metanalysis of five RCTs confirms previously suggested data, i.e., robotic surgery is associated with longer operating time and lower rate of conversions [6].

Data from the ROLARR trial however, to date the largest RCT published comparing laparoscopic and robotic rectal surgery, reported no differences in primary outcome – conversion rate, or secondary outcomes [13]. A follow-up on the ROLARR trial in 2018, adjusting for learning effects, suggested that the equality of outcomes seen in this study might have been influenced by the surgeons’ learning curve [28]. Surgeons in the laparoscopic group were proposed to be more experienced compared to surgeons in the robotic group, thus favoring the laparoscopic group. Balancing this notion are the reports that the learning curve for robotic surgery have been reported to be shorter for robotic compared to laparoscopy from several trials [9, 29].

Another intervention aiming to improve outcome from colorectal surgery has been ERAS protocols designed to reduce surgical stress. The ERAS protocol is proven to improve complication rates, shorten LOS and improve recovery [17] and recent studies also suggest that ERAS protocols may have a beneficial effect on long-term outcome [30, 31]. Although the use of evidence-based standardized perioperative interventions and careful measuring of adherence to the protocol facilitates comparison of surgical approaches, only a few studies comparing surgical approach within an ERAS protocol have been conducted. With the exception of the randomized controlled LAFA study [32] comparing laparoscopic and open colorectal surgery most studies are small single-center studies without enough power to detect differences in important variables. Only two of those studies are comparing robotic and laparoscopic rectal surgery [10, 11], both showing significantly lower postoperative complication rates, shorter LOS, lower conversion rates favoring robotic surgery.

In the current multicenter study, the difference in compliance to ERAS pre- and intraoperative interventions was small between groups, not only showing that ERAS interventions are feasible in all three surgical approaches, but also that the comparison is made on similar perioperative terms. Receiving a diverting ileostomy after rectal surgery could potentially affect LOS, but no difference in proportion of diverting stoma was shown between groups in the current study. In measuring postoperative compliance, variables that can also be considered as outcome variables important for mobilizing the patient, all were better in robotic vs open surgery. However, only two items, time to pain control and termination of urinary drainage, were better after robotic compared to laparoscopic surgery. On the other hand, results favored laparoscopy regarding passage of flatus/stool and time to tolerate solid food. Since there were no significant difference in symptoms delaying discharge and overall complications between robotic and laparoscopic surgery, time to pain control and termination of urinary drainage are the only variables measured that can explain the difference in length of stay in favor for robotic surgery. It could be argued that these two variables seem to be more important in predicting LOS than passage of stool and flatus and time to tolerate solid food. Lower conversion rate to open surgery in the robotic group most likely also contributed to shorter LOS. In this context, the fact that overall length of stay in the current study was rather long compared to a previous comparison within an ERAS setting needs attention [11]. We also acknowledge that the time patients meet criteria for discharge does not always match the time they actually leave hospital. This could have multiple causes, e.g., waiting for geriatric clinics to accept the patient, difficulties in handling a new stoma, high stoma output, etc.

Although overall complications were similar between groups in this study, the fact that open surgery had a lower rate of anastomotic leaks in univariate analysis may be found worrisome. However, techniques to detect leakage have improved over time and since the majority of open procedures were performed early in the study time period, this may affect the results. In addition, registration of leaks in EIAS most likely have improved over time. Also, increased use of preoperative radiotherapy later in the study period could have had an impact on leak rates. The lower conversion rate in the robotic group in this study was in line with previous publications and may be important since conversion rate to open surgery can be a proxy for difficult surgery [33] known to result in higher complication rates and worse oncological outcome [1, 34, 35]. Data on conversion from each individual hospital were too small given the low rate of conversion to yield any meaningful additional information. Although a significant difference in duration of surgery to the disadvantage of robotic vs laparoscopic surgery, this difference has to be considered as surprisingly small since many of the robotic procedures were performed during an early implementation phase of the technique. In the univariate analysis, preoperative chemo- and radiotherapy were more common in robotic surgery compared to laparoscopy, perhaps indicating more severe disease or a shift in treatment regimen. However, these variables were adjusted for in multivariate analysis.

The strength of the current study is the large sample size with prospectively and consecutively recorded data from several hospitals in Sweden which may reflect the true clinical reality to a larger extent compared to the study environment in many randomized studies. The ERAS protocol and the control over compliance also provides information that the same standardized perioperative care was given to all groups of patients which makes a comparison of different surgical techniques suitable since many perioperative variables always have an impact on the main clinical outcome and are seldom measured in other studies.

There are also limitations in this study. First, this study was not randomized. Using multivariate analysis to adjust for confounding and to avoid bias is not always enough to reach the level of gold standard. Second, we recognize that the 10-year inclusion time was long, with a larger proportion of laparoscopic and open operations in the beginning and robotic operations in the end of the study period. Although the results were adjusted for time in multivariate analysis and compliance were similar between groups over time, it may be difficult to fully correct for the fact that attitudes among the staff toward the ERAS program and different kind of technical advances may have changed over time resulting in better outcomes late in the study period. Third, many different hospitals were included in the study and the type of surgical approach were unequally distributed. Although the Swedish colorectal cancer registry shows similar outcome from surgery in different centers in Sweden, a possible impact on the results in the current study cannot be excluded. Fourth, the fact that EIAS does not include cancer stage is a drawback, since this variable, most likely, will influence outcome regardless of surgical approach.

In conclusion, this large multicenter cohort study within an ERAS setting showed overall better enhanced recovery outcome in robotic vs open surgery and lower conversion rates compared to laparoscopic surgery. With the exception of shorter LOS in the robotic group compared to the laparoscopic group no other differences in short-term outcomes were found. A combination of enhanced recovery programs and minimal invasive surgery continues to be beneficial for rectal cancer patients and merit further investigation to stimulate improvement.
